# Crispr-SGRU: Prediction of CRISPR/Cas9 Off-Target Activities with Mismatches and Indels Using Stacked BiGRU

**DOI:** 10.3390/ijms252010945

**Published:** 2024-10-11

**Authors:** Guishan Zhang, Ye Luo, Huanzeng Xie, Zhiming Dai

**Affiliations:** 1 College of Engineering, Shantou University, Shantou 515063, China; 2 School of Computer Science and Engineering, Sun Yat-sen University, Guangzhou 510006, China; 3 Guangdong Province Key Laboratory of Big Data Analysis and Processing, Sun Yat-sen University, Guangzhou 510006, China

**Keywords:** CRISPR/Cas9, off-target, stacked BiGRU, Deep SHAP, knowledge distillation

## Abstract

CRISPR/Cas9 is a popular genome editing technology, yet its clinical application is hindered by off-target effects. Many deep learning-based methods are available for off-target prediction. However, few can predict off-target activities with insertions or deletions (indels) between single guide RNA and DNA sequence pairs. Additionally, the analysis of off-target data is challenged due to a data imbalance issue. Moreover, the prediction accuracy and interpretability remain to be improved. Here, we introduce a deep learning-based framework, named Crispr-SGRU, to predict off-target activities with mismatches and indels. This model is based on Inception and stacked BiGRU. It adopts a dice loss function to solve the inherent imbalance issue. Experimental results show our model outperforms existing methods for off-target prediction in terms of accuracy and robustness. Finally, we study the interpretability of this model through Deep SHAP and teacher–student-based knowledge distillation, and find it can provide meaningful explanations for sequence patterns regarding off-target activity.

## 1. Introduction

CRISPR/Cas9 is a widely acclaimed gene editing technique and has been increasingly applied in various fields [1,2,3,4]. This system mainly consists of two main parts: a single guide RNA (sgRNA) for guiding and binding to target DNA, and the Cas9 endonuclease protein for cutting DNA at three base pairs (bp) upstream of the protospacer adjacent motif (PAM) [5]. Although CRISPR/Cas9 holds tremendous potential for both basic and clinical research, it presents a big challenge for off-target effects [6]. Previous studies have shown that 1–5 bp mismatches at the 5′ end of sgRNAs are tolerated, while an increased number of mismatches can lead to instability in gene targeting operation [7]. Off-target mutation is still one major concern when using CRISPR/Cas9 in biomedical and clinical applications [8]. Several experimental techniques have been developed to detect off-target sites [9,10]. However, they are limited in the ability to detect off-target sites with insertions or deletions (indels), due to inefficient post-processing procedures [11].

Many computational techniques have been proposed for off-target prediction. These methods can be roughly divided into two categories, namely, rule-based and learning-based. Rule-based methods typically score off-target effects based on the mismatch positions of guide sequences (e.g., CROP-IT [12]). Machine learning-based methods predict the off-target activity using algorithms by extracting various sequence-derived features [13], but they require domain knowledge to manually design the feature extractor [14]. Deep learning can automatically learn the intrinsic patterns from large-scale data. A convolutional neural network (CNN) [15] can capture the hierarchical spatial representation and local patterns in sequence data. A recurrent neural network (RNN) [16] is capable of processing input sequences of arbitrary length. A long short-term memory network (LSTM) [17,18], a special form of RNN, is designed to remedy the exploding or vanishing gradient problem in RNNs by introducing a memory cell. Gated recurrent unit (GRU) [19] is a variant of LSTM with fewer gating units. Bidirectional LSTM (BiLSTM) [20] and bidirectional GRU (BiGRU) can make use of context information of input sequence and capture long-range dependencies. Some off-target prediction methods have shown complementary strength by combining CNN and RNN, such as CRISPR-Net [21], which combines Inception [22] and BiLSTM; CRISPR-IP [23] and CRISPR-M [24], which incorporate CNN, BiLSTM, and attention [25]; and CrisprDNT [26], which incorporates CNN, BiLSTM, and transformer [27]. It is reasonable to apply a hybrid of CNN and RNN for off-target prediction because CNN performs well in learning the spatially invariant patterns, while RNN can retain information of the input sequence.

A traditional RNN has only one hidden layer, which may limit the performance for feature extraction [28,29]. Stacked RNNs are comprised of multiple hidden RNN layers, which allow the network to effectively model complex dependencies within a sequence [30]. Each RNN layer is regarded as a feature extractor, progressively transforming the input data into high-level representations. The features extracted by stacked RNNs are more extensive [31]. Several studies have shown that employing stacked RNNs can significantly improve the performance of feature extraction of sequences. For example, Chakraborty et al. [32] proposed a microRNA prediction model by incorporating CNN and stacked LSTM. The authors employed CNN to extract the features from sequences of mRNA and stacked LSTM to predict miRNA. The prior success of stacked RNN in bioinformatics inspired us to extend its application to off-target prediction.

Though numerous deep learning-based models have been developed for off-target prediction, few of them consider the indels between sgRNA–DNA sequence pairs. Several studies have shown that indels contribute significantly to off-target problems [33,34]. Specifically, off-target sites with indels close to the PAM are less likely to be active [10,11,35]. Thus, this calls for methods that can predict off-target activities with both mismatches and indels. Data imbalance issue is a noteworthy challenge for off-target prediction. Models trained on such data tend to be biased towards the majority class and thus perform poorly for the minority class [36]. How to deal with the data imbalance problem in the learning process of deep learning-based off-target prediction models is of value to explore. In addition, although existing methods can automatically learn the underlying representation of sequence pairs, there is still room for improving the interpretability.

To address the above limitations, we propose a hybrid Crispr-SGRU architecture, which combines an Inception and a stacked BiGRU, intended for predicting off-target activities with mismatches and indels. We suspect that multi-scale Inception is well suited for capturing local patterns at various scales, and stacked BiGRU is excellent for learning both short- and long-term dependencies of sgRNA–DNA sequence pairs. To the best of our knowledge, this is the first application of a stacked BiGRU for off-target prediction. Additionally, we use the dice loss function to address the data imbalance issue. Experimental results reveal our method outperforms existing methods in terms of accuracy and robustness. Furthermore, we use teacher–student-based knowledge distillation (KD) to assess the importance of base-pairing at specific positions and quantify these base pairs’ contributions using Deep SHAP. Our model exhibits superior performance in identifying meaningful sequence patterns regarding off-target activity, thereby furnishing researchers with more profound biological insights.

## 2. Results

### 2.1. Crispr-SGRU Can Accurately Predict Off-Target Activity

**Figure 1:**
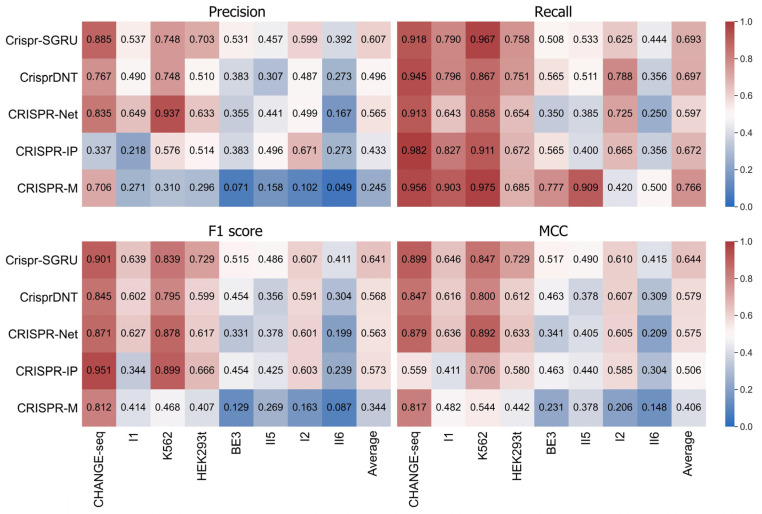
Heatmaps of precision, recall, F1 score, and MCC values of Crispr-SGRU and four existing methods on eight datasets with varying imbalance ratios. The predictors are placed vertically, whereas the test datasets are arranged horizontally. Datasets are sorted by imbalance ratio in ascending order.

We first assess whether our Crispr-SGRU can accurately predict off-target activity. We compare it with four existing hybrid CNN and BiLSTM methods, namely, CRISPR-Net [21], CrisprDNT [26], CRISPR-IP [23], and CRISPR-M [24], on eight public imbalanced datasets: CHANGE-seq, I1, K562, HEK293t, BE3, II5, I2, and II6. Prior to this, we briefly comment on some comparisons of these methods. First, CrisprDNT used hybrid one dimensional CNN and BiLSTM, whereas others applied two-dimensional CNN and BiLSTM. Second, besides CNN and BiLSTM, CRISPR-IP and CrisprDNT used attention mechanism and transformer, respectively. Third, CRISPR-M operated by incorporating both sgRNA–DNA sequence pairs and epigenetic data, whereas others considered only sgRNA–DNA sequence pair information. Thus, we compared these methods by considering sgRNA–DNA sequence pairs along to ensure fairness of the comparisons. Fivefold cross-validation is applied in the training and testing phases to achieve the averaged results. The quantitative results for each of the predictors are represented as heatmaps in Figure 1. We observe that models trained and tested on balanced datasets achieve superior performance than on imbalanced datasets. Our model achieves comparable performance to others in terms of recall (0.693) and outperforms them in terms of precision, F1 score, and MCC, with mean values of 0.607, 0.641, and 0.644, respectively. Crispr-SGRU shows excellent performance on the HEK293t dataset (IR = 247), with precision of 0.703, recall of 0.758, F1 score of 0.729, and MCC of 0.729. We also observe that Crispr-SGRU exhibits remarkable performance on the imbalanced II5 dataset (IR = 1773.6) and II6 dataset (IR = 6846.6) in terms of F1 score and MCC.

**Figure 2:**
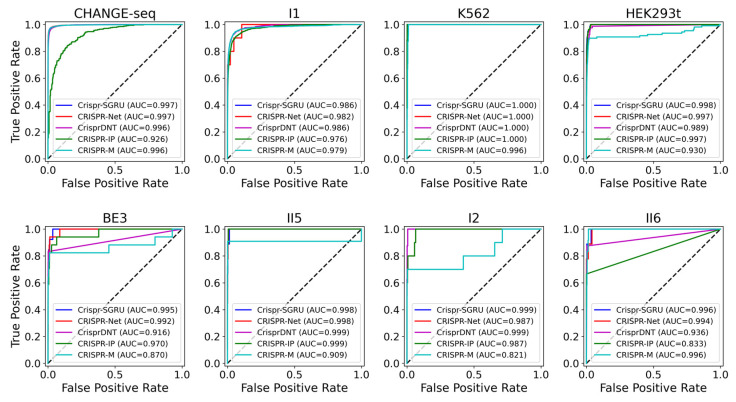
ROC plots for Crispr-SGRU and four deep learning-based methods on eight datasets under five-fold cross-validation.

**Figure 3:**
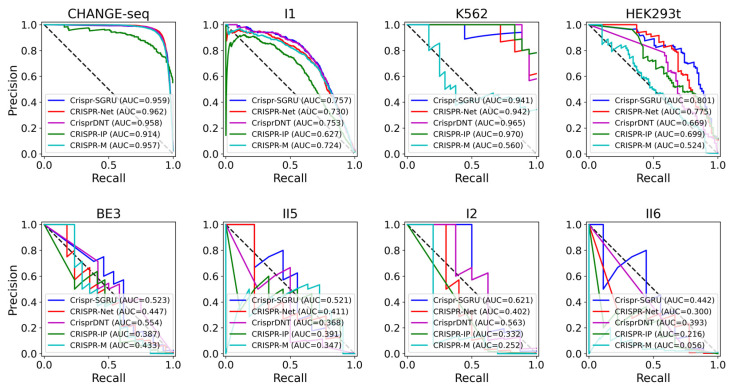
PR plots for Crispr-SGRU and four deep learning-based methods on eight datasets under five-fold cross-validation.

As shown in Figure 2 and Figure 3, our model outperforms the comparison models on both the AUROC and PRAUC evaluations. It shows the best performance with a PRAUC of 0.521 and 0.442 on imbalanced datasets II5 and II6, with a significant improvement of 11% and 4.9% compared with the second-best method. On dataset I1, our model outperforms other methods in terms of AUROC and PRAUC, with values of 0.986 and 0.757, respectively. Similarly, we observe that Crispr-SGRU remarkably suppresses other methods on datasets HEK293t, I2, and II6 in terms of these two evaluation indicators. Furthermore, our model outperforms CrisprDNT on datasets I1 and I2 on the measurement of PRAUC, with values increasing by 0.4% and 5.8% on average, respectively. For more details, see Supplementary Table S1. We also assessed the performance of our Crispr-SGRU on balanced datasets. For the sake of clarity, we compared it with two better methods, CRISPR-Net and CRISPR-IP as in Dhanjal et al., and II1 datasets under five-fold cross-validation, and the averages of the individual performances are summarized in Supplementary Table S2. Our Crispr-SGRU clearly outperformed the compared methods on balanced datasets. Collectively, these observations indicate that Crispr-SGRU outperforms competing methods.

### 2.2. Crispr-SGRU Can Robustly Predict Off-Target Activity

To assess the robustness of our model for off-target prediction, we compared it with the above four methods on datasets K562, HEK293t, BE3, and II5 under a leave-one-sgRNA-out procedure. Methods assessed by each sgRNA separately can reduce the risk of biasing the results towards sgRNAs with dominant evaluation calculation. For a fair comparison, we adopted the same dataset under a leave-one-sgRNA-out cross-validation procedure for model training and testing to avoid the impact of sgRNA selection. Specifically, for each dataset, we randomly selected the samples of an sgRNA as an independent testing set and the remaining sgRNAs and the corresponding sequence pairs served as a training set in each iteration. To assess the performance on independent datasets, for each sgRNA, all methods were trained on the rest of this dataset, excluding the dataset containing this sgRNA. This approach can guarantee that the training set and testing set have no sgRNA overlap. Supplementary Table S3 shows the details of the selected sgRNAs and the number of positive and negative samples in each dataset.

**Figure 4:**
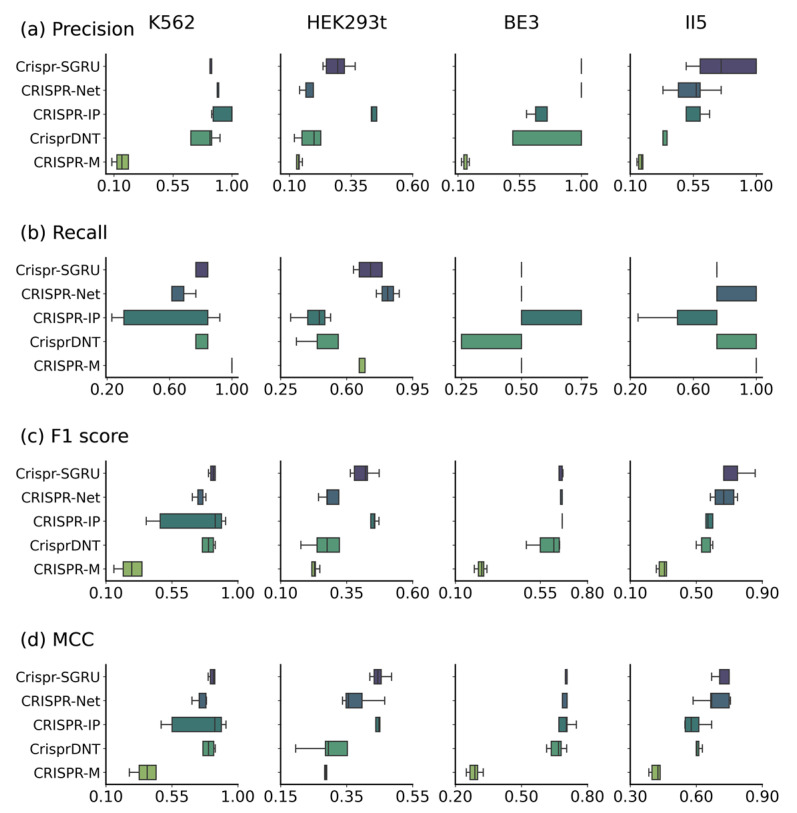
Performance comparison of Crispr-SGRU with four other methods (e.g., CRISPR-Net, CRISPR-IP, CrisprDNT, and CRISPR-M) on datasets K562, HEK293t, BE3, and II5 under a leave-one-sgRNA-out test.

**Figure 5:**
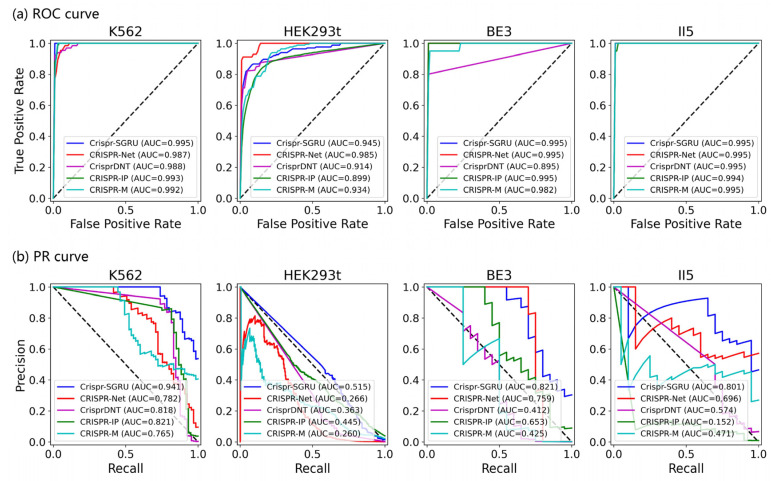
ROC and PR plots of Crispr-SGRU with four predictors (CRISPR-Net, CRISPR-IP, CrisprDNT, and CRISPR-M) implemented on four datasets—K562, HEK293t, BE3, and II5—under a leave-one-sgRNA-out test.

As Figure 4 and Figure 5 depict, Crispr-SGRU shows excellent performance on the K562 dataset, with an average F1 score of 0.828 and MCC of 0.826. It achieves comparable performance to CrisprDNT, with averaged value of 0.880 and 0.910 in terms of all evaluation metrics. Our model significantly surpasses CrisprDNT on the HEK293t dataset based on precision, recall, F1 score, MCC, and PRAUC measurements, with improvements of 0.9%, 23%, 4.1%, 6.9%, and 20%, respectively. In addition, Crispr-SGRU has the second-highest AUROC (0.945), about 4% lower than CRISPR-Net on this dataset. Crispr-SGRU achieves the best averaged AUROC (0.999), PRAUC (0.895), and MCC (0.695) on the BE3 dataset, with a 4% improvement in PRAUC and a 1.4% improvement in MCC over the second-best method. Moreover, it achieves the highest average F1 score (0.721) and MCC (0.740) on the II5 dataset. This corresponds to a 5.2% improvement in F1 score and a 5.4% enhancement in MCC over the second-best method. Together, these results suggest that our method surpasses others in terms of robustness.

### 2.3. Ablation Analysis Shows the Importance of Stacked BiGRU

We evaluated the effectiveness of different network architectures for Crispr-SGRU on off-target prediction. To this end, we carried out a comparative analysis of two variants derived from our final model by modifying its components. First, we verified the efficacy of the Inception by proposing a variant architecture (Crispr without Inception), getting rid of the Inception layer from the full model. We directly used the output of encoded sequences as inputs for the stacked BiGRU instead. Second, we confirmed the efficacy of the stacked BiGRU by constructing a variant architecture (Crispr without BiGRU) by removing the stacked BiGRU. We used the flattened output of the Inception layer as the input of the dense layer. Then, we compared these variants with Crispr-SGRU under five-fold cross-validation on the K562 and HEK293t datasets.

The experimental results are shown in Table 1. We observed that these two variants performed significantly worse than the final model, suggesting that both Inception and stacked BiGRU are essential. Furthermore, we discovered that removing Inception layers can obviously degrade the performance metrics. This proves the effectiveness of multi-scale convolutional operations in detecting the abstract sequence features. Notably, we observed that eliminating the stacked BiGRU module resulted in a significant decrease in all performance metrics across these two datasets, leading to 19.7% and 4.8% decreases on average on the K562 and HEK293t datasets, respectively. Thus, the stacked BiGRU is indispensable in our model for its ability to capture the rich sequence dependencies and contributes most to off-target prediction.

### 2.4. Crispr-SGRU Can Effectively Make Off-Target Activity Prediction

Next, we compared the computational efficiency of Crispr-SGRU and four deep learning-based methods—CRISPR-Net, CrisprDNT, CRISPR-IP, and CRISPR-M—on the HEK293t dataset. The batch size and epoch were set to 256 and 30, respectively. The running time results can be found in Supplementary Table S4. Overall, our method demonstrates a certain level of advantage in efficiency among all methods. We also observe that CrisprDNT requires the most time to execute. This is expected, because CrisprDNT utilizes a transformer module, which inherently possesses high computational complexity. In contrast, CRISPR-IP introduces a single-layer attention mechanism, which runs seven times faster than CrisprDNT. These results illustrate that though deep networks can better capture the characteristics of data, the computational cost of the model increases exponentially as the number of hyperparameters increases. Thus, there is a trade-off between performance and computational cost.

### 2.5. Model Interpretability

**Figure 6:**
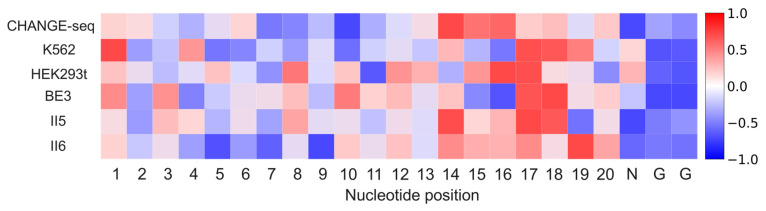
Visualization of the importance of various positions for Crispr-SGRU on six mismatch-only datasets. The color of each cell in the heatmap represents the contribution of nucleotide positions to the off-target prediction.

We were also interested in the interpretability of our model. We applied Deep SHAP [37] to estimate the position-dependent nucleotide contribution to our model on six mismatch-only datasets: CHANGE-seq, K562, HEK293t, BE3, II5 and II6. For each dataset, we used all positive samples and randomly selected an equal number of negative samples for analysis. To make the contribution of nucleotides comparable among various datasets, we rescaled the SHAP values by standardization. As depicted in Figure 6, nucleotides at positions 10–20 exhibit a strong positive contribution to off-target prediction on all datasets. This result aligns with previous studies, which corroborate that nucleotides at 8–12 positions adjacent to the PAM determine Cas9 specificity [38,39,40]. In addition, we observe the nucleotide at position 18 has a positive contribution on all datasets, which is in line with previous findings that nucleotides at positions 2–3 distal to the PAM are less tolerant of mismatches [41,42]. Our model consistently pays close attention to positions 1–3. This observation is in accordance with previous findings, suggesting that mismatches at positions 1–3 are less likely to be tolerated [42]. We also find that positions 1–5 have a positive contribution on CHANGE-seq, K562, HEK293t, and II5 datasets. These observations are in agreement with Vora et al. [40], which demonstrates that the PAM-distal-most positions provide valuable information.

Supplementary Table S5 shows the performance of Crispr-SGRU and two student models: the student model with KD (Student with KD) and student model without KD (Student without KD) on K562 dataset under fivefold cross-validation. Compared to Student without KD, Student with KD boosts the performance in terms of all evaluation metrics, with AUROC, PRAUC, F1 score, and MCC increased by 0.2%, 3.1%, 1.1%, and 4.6%, respectively. This observation illustrates that knowledge is successfully transferred from the teacher model to the student model. Thereby, Student with KD can be applied to interpret the decision basis of Crispr-SGRU.

**Figure 7:**
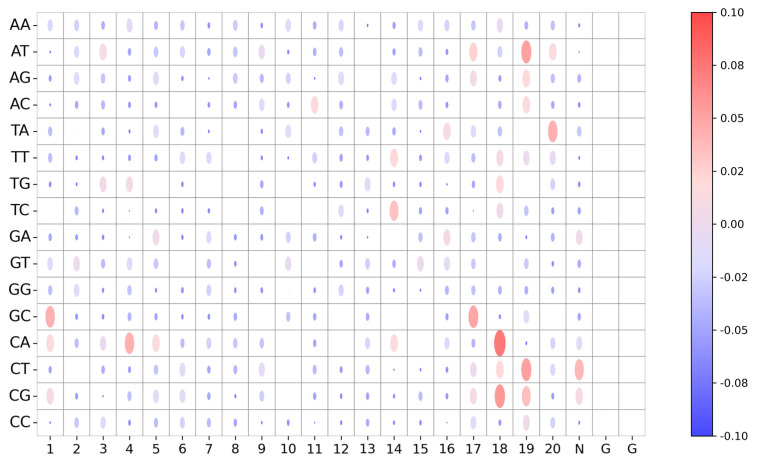
Visualization of importance of the sequence context for each position in sgRNA–DNA sequence pairs for Student with KD model on K562 dataset. The nucleotide positions are arranged horizontally, whereas various sequence contexts are placed vertically. Color and size of the dots in the bubble plot represent the average Deep SHAP values, which represent the contribution of nucleotide position to off-target prediction.

To improve the reliability and transparency of decision-making in the model and reveal the role of the stacked BiGRU in the student model, we visualize the model weight of Student with KD of each base-pairing of an sgRNA–DNA sequence pair and analyze the important sequence fragments obtained from the model. We apply Deep SHAP to visualize the model weight maps for 16 base pairs for each position in sgRNA–DNA sequence pairs for Student with KD. Figure 7 shows the importance of the sequence context for each position in sequence pairs for Student with KD on K562 dataset. We observe base-pairing at positions 14–20 achieve higher Deep SHAP values, which means these positions have significant influence on prediction among all sites of sequence pairs. Specifically, C-A:18 (representing the mismatch site C-A at position 18) has the highest SHAP value, which means it has the most significant positive impact on off-target prediction. We also find that the mismatch sites A−T:19, C−T:19, C−G:19, and T−A:20 have great effect on predictive outcomes. This phenomenon is expected, since CRISPR/Cas9 specificity depends on the sgRNA seed sequence of the PAM-proximal region [43]. In addition, G−C:17, C−G:18, and T−G:18 are influential, with positive contributions to prediction. This is consistent with previous observations that the cut site-adjacent sequence is an important determining factor for Cas9-induced mutations [44,45,46]. Furthermore, we observe that some mismatch sites of the PAM-distal region, such as G−C:1, C−A:4, and C−A:5, have great influence on off-target prediction, which coincides with a previous finding that mismatches at positions 1–3 are less likely to be tolerated [42]. Together, these results indicate that our model can learn critical sequence information in sequence pairs for off-target prediction.

## 3. Discussion

CRISPR/Cas9 is a novel genome editing tool, but it also leads to unintended off-target sites. Recent studies have shown that off-target cleavage can potentially occur anywhere in genomes as long as the region contains a PAM and a protospacer sequence with base mismatches and indels [33]. Lin et al. [21] found that using both indel and mismatch sgRNA–DNA sequence pairs as input outperformed models using mismatch sgRNA–DNA sequence pairs only, which implies that the indels are relevant and should be considered in off-target prediction. In this work, we propose Crispr-SGRU, a deep learning framework that incorporates Inception and stacked BiGRU for predicting CRISPR/Cas9 off-target activities with mismatches and indels. We compare our model with four existing methods—CrisprDNT, CRISPR-Net, CRISPR-IP, and CRISPR-M—on eight public datasets. To further investigate the robustness of our model on unseen data, we compare it with other methods on four mismatch-only datasets using a leave-one-sgRNA-out approach. Our results show that Crispr-SGRU exhibits competitive performance in terms of accuracy and generalizability. Additionally, we introduce Deep SHAP to visualize the position-dependent nucleotide contribution to Crispr-SGRU. As expected, the visualization results show our model can focus on sgRNA seed sequence, which is important for off-target prediction. Moreover, we apply knowledge distillation in the framework by transferring the learnable information from the teacher model to the student model to identify informative sequence patterns regarding off-target activity. The prediction result of Crispr-SGRU and position-dependent mismatch-tolerance information can help the design of optimum sgRNA sequences for minimum chances of off-targeting. Validations of the current study are mainly performed in silico, and experimental validation is to be performed in the future to make the results more convincing. This study considered only off-targets with NGG PAMs. Apart from the canonical NGG PAM, Cas9 has been reported to cut off-target sequences with alternative PAM sequences (e.g., NAG and NGA) [21]. It is worth considering various PAM sequences in future work. Accurate prediction of off-target sites in CRISPR genome editing is highly important due to their potential disruptive effect and thus helps in sgRNA design with few off-target sites. We believe that our model can contribute to future research on the basic mechanisms underlying off-target cutting in CRISPR/Cas9, thus assisting the basic and clinical application of this technology.

## 4. Materials and Methods

### 4.1. Datasets

We used eight public datasets to evaluate the performance of our model. These datasets can be divided into two categories: (i) mismatch-only datasets (e.g., Dhanjal et al. [47], II1 [48], CHANGE-seq [49], K562 [50], HEK293t [50], BE3 [51], II5 [52], and II6 [53]), and (ii) mismatch-and-indel datasets (e.g., I1 [21] and I2 [21]). Each entry in these datasets is composed of an sgRNA sequence, a target DNA sequence, and a binary label, with 0 representing the negative sample and 1 representing the positive sample. All datasets contain only off-target sites with NGG PAMs. Dhanjal et al.’s dataset consists of 9214 positive samples and 9917 negative samples. The II1 dataset consists of 2273 positive samples and 2580 negative samples. These two datasets are balanced, with imbalance ratios of 1.10 and 1.14, respectively. The CHANGE-seq dataset contains 67,476 positive samples and 2,806,151 negative samples. The HEK293t and K562 datasets contain 18 and 12 sgRNAs, respectively. Among all 30 sgRNAs, 656 samples have been identified as off-targets. The HEK293t dataset consists of 536 positive samples and 132,378 negative samples. The K562 dataset contains 120 positive samples and 20,199 negative samples. The BE3 dataset contains 9 sgRNAs accounting for 79 validated off-targets [51]. The II5 dataset has 95,775 negative samples, while the number of positive samples is only 54. The I1 dataset consists of 10 sgRNAs, with a total of 7371 active off-targets, 340 with 1 bp indel and up to 3 bp mismatches and 7031 with up to 6 bp mismatches. Dataset I2 comprises 213,943 sequence pairs from 6 sgRNAs. This dataset is comprised of 60 validated off-targets, of which 13 contain indels. The II6 dataset contains 52 positive samples and 383,407 negative samples. More details are shown in Table 2.

### 4.2. Sequence Encoding

We use the one-hot encoding scheme proposed by Lin et al. [21] to encode sgRNA–DNA sequence pairs, where the DNA or RNA bulge (indel) is denoted using underscores. Each sequence pair is encoded as a seven-row binary matrix, with a five-bit channel (e.g., A, G, C, T, and indel) to encode the sgRNA and DNA nucleotides and a two-bit direction channel to identify the mismatch and indel directions. Specifically, sgRNA and DNA sequences are encoded by one-hot encoding, subsequently being performed by OR operation to get the results of the first four channels (e.g., AA (1, 0, 0, 0), TT (0, 1, 0, 0), GG (0, 0, 1, 0), CC (0, 0, 0, 1)). The fifth channel marks the DNA or RNA bulge, where indel occurs is 1, otherwise it is 0. The sixth and seventh channels are applied to identify the insertion or deletion and the mismatch type. To adapt the indel information in this encoding scheme, we add 1 nt using hyphens beside the target sequence and the PAM sequence to form a 24 nt base sequence or base pair sequence, which is encoded by (0, 0, 0, 0). As such, each sequence pair is encoded by a binary matrix with a size of (length of the sequence pair) × 7. We direct readers to the original publication [21] for more details.

### 4.3. Crispr-SGRU

**Figure 8:**
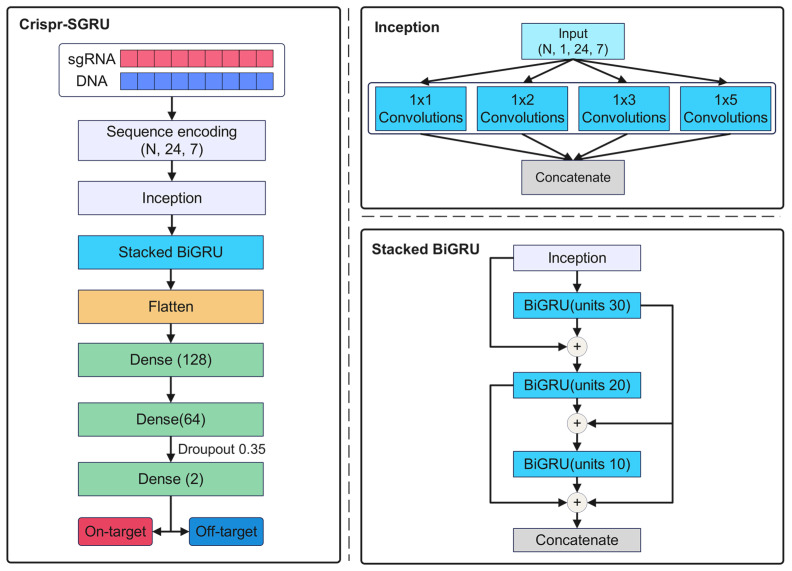
(**a**) The structure of Crispr-SGRU. Each sgRNA–DNA sequence pair is encoded and fed into an Inception module (**b**) for feature extraction. The outputs of Inception are put into the stacked BiGRU (**c**) to learn the sequential dependencies of the sequence pairs. The number of units in each BiGRU is 30, 20, and 10. The outputs of stacked BiGRU layers are concatenated and passed through three dense layers with 128, 64, and 2 neurons, respectively. A sigmoid activation function is applied in the output layer to obtain the final prediction results.

Our Crispr-SGRU model (Figure 8a) has two parts: an Inception-based CNN (Figure 8b) and a stacked BiGRU (Figure 8c). The CNN module is regarded as a feature extractor that learns the local and global features of sequence pairs independently. The stacked BiGRU is designed to recognize sequential patterns. Each sequence pair is encoded into a binary matrix, subsequently being fed into a CNN. The CNN module consists of four two-dimensional convolutional layers with varying filter sizes: 1 × 1, 1 × 2, 1 × 3, and 1 × 5, respectively. The units of these layers are set to 10. Utilizing varying perceptual fields can combine feature information at various scales during parallel processing [55]. This enables the internal layer to choose the suitable filter size for learning the required information of the sequences. Additionally, the parallel pooling operations in Inception can reduce the dimensions of the feature map. The outputs of Inception are input into the stacked BiGRU, which consists of three BiGRU layers of 30, 20, and 10 units, to learn the contextual information of the sequence. This is inspired by previous studies that stacked multiple RNN layers and extracted more generalized sequential features [32,56,57]. The outputs of the stacked BiGRU are flattened and fed into two dense layers with 128 and 64 units. The rectified linear unit (ReLU) [58] is applied as the activation function. We use a 0.35 dropout rate for model regularization to combat overfitting. The output layer consists of two neurons using a sigmoid activation function that quantifies propensity for on- and off-target sites. We use an Adam optimizer [59] with learning rate of 0.0001 to train our model to minimize the loss function.

### 4.4. Class-Balanced Loss Function

We regarded the off-target prediction as a binary classification task. This is inspired by a previous study that off-target assessment applying classification outperforms regression [50]. Several class-balanced loss functions have been proposed to solve the problem of training from imbalanced data by introducing a weighting factor inversely proportional to the effective number of samples [36], such as focal loss [60], Tversky loss [61], asymmetric loss [62], dice loss [63], and hinge loss [64]. The formulas of these loss functions are summarized in Supplementary Table S6. Inspired by previous work [26], we focused on the loss function to address the data imbalance problem while training our model. We compared the performance of Crispr-SGRU trained using the abovementioned loss functions and observed that using dice loss achieves superior performance to others (Supplementary Table S7). Thereby, we used dice loss in our model to reduce the impact of favoring the negative samples.

### 4.5. Knowledge Distillation Helps to Reveal Model Visualization Capability

KD [65] is applied to transfer the dark knowledge learned by the larger teacher model to a small student model. Some studies have also used this strategy in training to improve model interpretability. For instance, Lu et al. proposed a KD-insight drug–target affinity prediction method, which takes atomic-level pockets as input and learns drug–target atomic-level interaction information, thus giving the model better interpretability [66]. Fan et al. developed a deep learning-based method called ETFC to predict 21 categories of therapeutic peptides [67]. They constructed a student model with KD to investigate the contribution of each AA in the ETFC to each peptide sequence and enhanced the interpretability of their model. We note that sequence pairs are encoded by the encoding scheme proposed by Lin et al. [21] in our model, so the base-pairing information in each sequence pair is inevitably disrupted by an OR operator, which means our model lacks decision-making transparency. Inspired by the above studies, we constructed the KD-insight off-target prediction model, which takes a 16-channel encoded matrix as input and investigates the contribution of each base pair to each sgRNA–DNA sequence pair, thus giving the model better interpretability.

**Figure 9:**
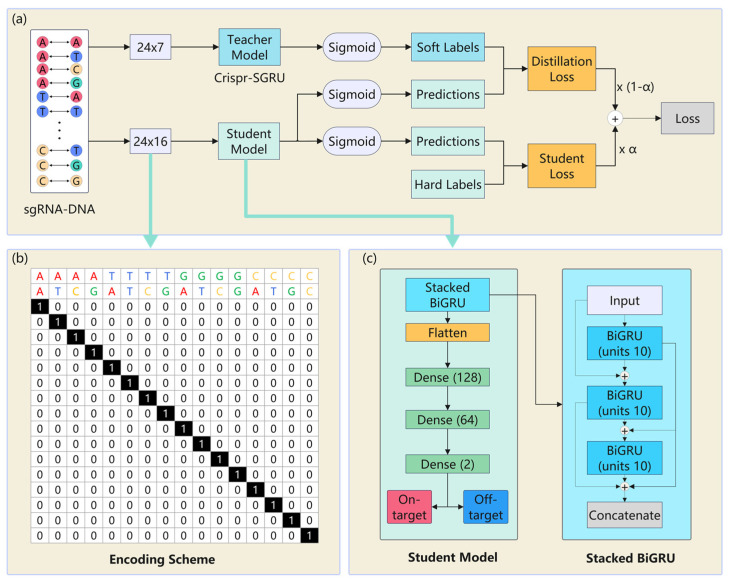
The teacher–student framework for knowledge distillation. (**a**) Workflow of knowledge distillation. Crispr-SGRU is the teacher model. The student model is applied to increase the interpretability of Crispr-SGRU. (**b**) Overview of the encoding scheme and (**c**) student model.

Figure 9a shows the teacher–student workflow for KD. We defined the pretrained Crispr-SGRU as the teacher model, while the student model is based on it with fewer parameters. The details and the encoding scheme and student model are exhibited in Figure 9b,c. We designed a 24 × 16 encoding scheme to encode sequence pairs, where 24 represents the length of the sequence pairs. For an sgRNA–DNA sequence pair, the base pair is represented by 16 channels, i.e., AA-, AT-, AC-, AG-, TA-, TT-, TC-, TG-, GA-, GT-, GC-, GG- CA-, CT-, CG-, CC-, and CG-. Each position in each sequence pair is related to a vector of length 16 with a single non-zero element corresponding to the base pair at that position. For example, AA and CG are represented as vectors [1, 0, 0, 0, 0, 0, 0, 0, 0, 0, 0, 0, 0, 0, 0, 0] and [0, 0, 0, 0, 0, 0, 0, 0, 0, 0, 0, 0, 0, 0, 0, 1], respectively. This encoding scheme can preserve the information of both sequence and mismatch types, thus avoiding loss of important information during model training and enabling accurate interpretation of the base-pair details.

In the KD process, we trained the student model with real sample labels as hard labels, which typically convey information about the predicted classes. The predictive outcomes of the teacher model were regarded as soft labels that could offer more comprehensive insights and salient knowledge derived from the probability distribution across all classes during training the student model. We utilized the KD model to extract more detailed information from the teacher–student model and obtained refined soft labels using the student model. We employed distillation loss (DL) to quantify the loss incurred during model training. We used student loss (SL) to denote the loss incurred during this process. Thus, the loss function of the student model considered the distribution of labels from the teacher model, which is defined as follows:

(1) $$L_{KD}=\alpha *SL+(1-\alpha )*DL$$

where α is a hyperparameter used to balance the losses of SL and DL. We set α=0.2 in the following experiment.

### 4.6. Experimental Setup

Crispr-SGRU was performed using Python 3.8 and Keras library 2.4.3 with a Tensorflow (2.5.0) backend. All experiments were carried out on a desktop computer with an Intel (R) Xeon (R) Silver 4210R CPU (Intel, Senta Clara, CA, USA) @ 2.40 GHz, Ubuntu 18.04.6 LTS, and one NVIDIA GeForce RTX 3080 Ti with 12 GB of memory (MSI, New Taipei, China). For each dataset, we adopted the standard data split: 85% and 15% for model training and testing, respectively. We trained the models with a batch size of 256 and epoch of 30. Additionally, we applied the bootstrapping sampling algorithm [68] to ensure that each batch had equivalent positive samples and negative samples in the training data, thus preventing gradient update instability during model training.

### 4.7. Evaluation Metrics

To assess model accuracy, we used four commonly used evaluation metrics—precision, recall, F1 score [69], and Matthews correlation coefficient (MCC) [70]—which are defined as follows:

(2) $$Precison=\frac{TP}{TP+FP}$$

(3) $$Recall=\frac{TP}{TP+FN}$$

(4) $$F1score=\frac{2\times Precision\times Recall}{Precision+Recall}$$

(5) $$MCC=\frac{TP\times TN-FN\times FP}{\sqrt{\left(TP+FN\right)\times \left(TN+FP\right)\times \left(TP+FP\right)\times \left(TN+FN\right)}}$$

where TP is the number of correctly predicted off-targets, FN is the number of off-targets predicted to be non-off-targets, TN is the number of correctly predicted non-off-targets, and FP is the number of non-off-targets predicted to be off-targets. The F1 score offers a balanced measurement by considering both precision and recall, thus providing insights into both accuracy and completeness of the model. Additionally, we constructed receiver-operating characteristic (ROC) [71] and precision–recall (PR) curves [72] to visualize model ability and calculated the corresponding area under the ROC curve (AUROC) and area under the PR curve (PRAUC) to quantify the model’s performance. AUROC can better evaluate the prediction performance when the labels are highly imbalanced, which is the case for off-target prediction.

## Figures and Tables

**Table 1:** Performance comparison of Crispr-SGRU and its two variants (Crispr without Inception and Crispr without BiGRU) on K562 and HEK293t datasets under fivefold cross-validation.

Model	Precision	Recall	F1 Score	MCC	AUROC	PRAUC	Mean
(a) K562							
Crispr-SGRU	0.802	0.922	0.821	0.856	1.000	0.928	0.888
Crispr without Inception	0.744	0.867	0.793	0.800	0.999	0.922	0.854
Crispr without BiGRU	**0.561**	**0.656**	**0.600**	**0.602**	**0.998**	**0.730**	**0.691**
(b) HEK293t							
Crispr-SGRU	0.697	0.704	0.700	0.705	0.998	0.780	0.764
Crispr without Inception	0.673	0.691	0.696	0.696	0.998	0.771	0.754
Crispr without BiGRU	**0.644**	**0.654**	**0.648**	**0.647**	**0.996**	**0.707**	**0.716**

Note: Crispr without Inception is a variant that does not use the Inception module. Crispr without BiGRU is a variant that does not use the stacked BiGRU block. The worst performance as measured by each metric across different architectures are respectively highlighted in bold for clarification.

**Table 2:** Details of the off-target datasets used in our study.

Dataset	Cell Type	Positive	Negative	Imbalance Ratio	Category	Ref.
Dhanjal et al.	Human cell line	9214	9917	1.10	Mismatch	[47]
II1	A357, HT29, 293T	2273	2580	1.14	Mismatch	[48]
CHANGE-seq	Human primary T cells	67,476	2,806,151	41.6	Mismatch	[54]
I1	U2OS, HEK293t, K562, PGP1	7371	577,577	78.4	Mismatch and indel	[21]
K562	K562	120	20,199	168.3	Mismatch	[50]
HEK293t	HEK293t	536	132,378	247.0	Mismatch	[50]
BE3	HEK293t	83	93,434	1125.7	Mismatch	[51]
II5	EGFP, U2OS	54	95,775	1773.6	Mismatch	[21]
I2	U2OS	50	231,883	4277.7	Mismatch and indel	[21]
II6	HCT116, HEK293t, HL60, Kbm7, K562, U2OS	52	383,407	6846.6	Mismatch	[21]

## Data Availability

The original contributions presented in the study are included in the article/Supplementary Material. Further inquiries can be directed to the corresponding author.

## Supplementary Materials

The following supporting information can be downloaded at https://www.mdpi.com/article/10.3390/ijms252010945/s1.
