# Supplementary material for: Crispr-SGRU: Prediction of CRISPR/Cas9 Off-Target Activities with Mismatches and Indels Using Stacked BiGRU
Source: Int J Mol Sci. 2024 Oct 11;25(20):10945. doi: 10.3390/ijms252010945 (PMC11507390; doi:10.3390/ijms252010945)
Supplement: Supplementary file 1 [file ijms-25-10945-s001.zip › ijms-3225323-supplementary.pdf]

Supplementary Table S1. Performance comparisons amongst five deep learning-based methods on eight datasets under five-fold cross-validation

Method	CHANGE-seq	I1	K562	HEK293t	BE3	II5	I2	II6
(a) AUROC								
Crispr-SCRU	**0.997**	**0.986**	**1.000**	**0.998**	**0.995**	0.998	**0.999**	**0.996**
CRISPR-Net	**0.997**	0.982	**1.000**	0.997	0.992	0.998	0.987	0.994
CrisprDNT	0.996	**0.986**	**1.000**	0.989	0.916	**0.999**	**0.999**	0.936
CRISPR-IP	0.926	0.976	**1.000**	0.997	0.970	**0.999**	0.987	0.833
CRISPR-M	0.996	0.979	0.996	0.930	0.870	0.909	0.821	**0.996**
(b) PRAUC								
Crispr-SCRU	0.959	**0.757**	0.941	**0.801**	0.523	**0.521**	**0.621**	**0.442**
CRISPR-Net	**0.962**	0.730	0.942	0.775	0.447	0.411	0.402	0.300
CrisprDNT	0.958	0.753	0.965	0.669	**0.554**	0.368	0.563	0.393
CRISPR-IP	0.914	0.627	**0.970**	0.699	0.387	0.391	0.332	0.216
CRISPR-M	0.957	0.724	0.560	0.524	0.433	0.347	0.252	0.056

Note: The top table records AUROC values while the bottom one records PRAUC values. The best performance as measured by each metric across different methods is highlighted in bold for clarification. These highlights also apply to Supplementary Tables 2 and 5.

Supplementary Table S2. Performance comparison of Crispr-SGRU and other two deep learning-based methods on two balanced datasets (e.g., Dhanjal et al. dataset and II1 dataset) under five-fold cross-validation

Method	Precision	Recall	F1 score	MCC	AUROC	PRAUC	Average
(a) Dhanjal et al.							
Crispr-SGRU	0.958	**0.824**	**0.867**	**0.748**	0.951	0.958	**0.884**
CRISPR-Net	0.948	0.559	0.713	0.618	0.926	0.948	0.785
CRISPR-IP	**0.970**	0.689	0.809	0.710	**0.965**	**0.970**	0.852
(b) II1							
Crispr-SGRU	**0.477**	0.817	**0.597**	**0.497**	**0.892**	**0.735**	**0.669**
CRISPR-Net	0.333	**0.883**	0.481	0.354	0.794	0.508	0.559
CRISPR-IP	0.311	0.867	0.422	0.271	0.840	0.618	0.555

Supplementary Table S3. Details of randomly selected sgRNAs from four datasets using leave-one-sgRNA-out approach

Dataset	sgRNA	Positive	Negative
K562	GGTCCTGCCGCTGCTTGTCATGG	13	729
HEK293t	GCCTCTTTCCCACCCACCTTGGG	33	2714
BE3	GGCCCAGACTGAGCACGTGATGG	4	8327
II5	GTGCGGCAAGAGCTTCAGCCAGG	4	6862

Supplementary Table S4. The running time of the comparison between Crispr-SGRU and four off-target prediction methods on the HEK293t dataset

Model	Time
Crispr-SGRU	1497
CRISPR-Net	2191
CrisprDNT	10791
CRISPR-IP	1556
CRISPR-M	2580

Supplementary Table S5. Performance comparison among Crispr-SGRU and two student models (i.e., Student w/KD and Student w/o KD) on K562 dataset under five-fold cross-validation

**Model**	**AUROC**	**PRAUC**	**F1 score**	**MCC**	**Average**
Crispr-SGRU	**1.000**	**0.928**	**0.821**	**0.856**	**0.901**
Student w/KD	1.000	0.910	0.811	0.840	0.890
Student w/o KD	0.998	0.879	0.800	0.794	0.868

Note: Student w/KD is the student model applying KD. Student w/o KD is the student model without applying KD.

Supplementary Table S6. An overview of the loss functions used for data imbalance issue

Loss function	Formula	Hyperparameter	Ref
Focal	$Focal = -\alpha(1-p)^{\wedge}\beta * log(p)$	$\alpha = 0.25, \beta = 0.75$	[62]
Tversky	$Tversky = \frac{TP}{TP + u + FP + v + FN}$	$u = 0.7, v = 0.3$	[63]
Asymmetric	$Asymmetric = \alpha * (1-y) * \log(p) + (1-\alpha) * y * \log(1-p)$	$\alpha = 0.5$	[64]
Dice	$Dice = 1 - \frac{2 * TP}{2 * \alpha * TP + \beta * FP + \beta * FN}$	$\alpha = 0.7, \beta = 2$	[65]
Hinge	$Hinge = \max(0, 1 - y * p)$	-	[66]

Note: TP represents true positive, FP denotes false positive, and FN means false negative. Besides, p denotes the predicted value and y refers to the true label. α represents the balancing factor used to balance the weights of positive samples and negative samples. β means the modulating factor to control the attention level of difficult samples. Values of u and v are applied for balancing the weights of FP and FN.

Supplementary Table S7. Effect of various loss functions for Crispr-SGRU on K562 and HEK293t datasets

**Loss function**	**F1 score**	**MCC**	**AUROC**	**PRAUC**	**Average**
(a) K562					
Dice	***0.839***	***0.847***	***1.000***	***0.932***	***0.905***
Focal	0.778	0.782	0.998	0.873	0.858
Tversky	0.743	0.743	0.997	0.843	0.832
Asymmetric	0.767	0.769	0.999	0.871	0.852
Hinge	0.680	0.681	0.998	0.807	0.792
(b) HEK293t					
Dice	***0.729***	***0.729***	***0.998***	***0.787***	***0.811***
Focal	0.723	0.723	***0.998***	0.782	0.807
Tversky	0.597	0.612	0.997	0.686	0.723
Asymmetric	0.637	0.641	0.996	0.703	0.744
Hinge	0.655	0.659	0.994	0.721	0.757

Note: The best performance as measured by each metric are respectively highlighted in bold and italic for clarification.
